# Storage of Cereals in Warehouses with or without Pesticides

**DOI:** 10.3390/insects11120846

**Published:** 2020-11-28

**Authors:** Darka Hamel, Vlatka Rozman, Anita Liška

**Affiliations:** 1 Croatian Agency for Agriculture and Food-Centre for Plant Protection, 10000 Zagreb, Croatia; 2 Faculty of Agrobiotechnical Sciences Osijek, Josip Juraj Strossmayer University of Osijek, 31000 Osijek, Croatia; vrozman@fazos.hr (V.R.); aliska@fazos.hr (A.L.)

**Keywords:** integrated pest management, cereals, insects, quality, damage, storage

## Abstract

**Simple Summary:**

For decades, the use of various synthetic pesticides has been the key factor in the proper and long-term storage of cereals. Unfortunately, we are faced with non-acceptable data regarding the effects of synthetic pesticides. Due to this, further steps have been made in order to take measures to reduce the use and risk of chemical pesticides by 50% by 2030 and to reduce the use of more dangerous pesticides by 50% by 2030. The concept of integrated pest management has been promoted as a dynamic and flexible approach leading to the reduction of chemical pesticide usage and their negative effects on the environment. The aim of this review is to indicate how cereals stored in silos or warehouses are handled and what measures are taken to preserve them, to describe the situation regarding pesticides, and to point out the problems occurring during application and the possibility of applying substitutions. It has to be taken into account that many of these measures cannot completely control insect or mite populations and are very demanding because of the need for more knowledge and experience, better equipment, greater financial investment, and a higher awareness of the impacts of pesticides not only for agricultural producers and storage keepers, but also for consumers.

**Abstract:**

At a time when there is much talk of reducing pesticide use and the implementation of integrated pest management, mainly in fields and glass-houses, it is appropriate to consider how cereals in storage are handled and what measures are taken to protect them against insects and other pests. For decades, the use of various synthetic pesticides has been the basis for the proper and long-term storage of cereals, primarily free of insects and mites, but also fungi and their mycotoxins and rodents. However, due to the registered negative effects of synthetic pesticides, such as dichloro-diphenyl-trihloroethane (DDT) or methyl bromide, on human health and the environment, and the appearance of resistance to, e.g., malathion, researchers have been looking for new acceptable control measures. Due to the proven and published non-acceptable data regarding synthetic pesticide effects, a combination of physical, mechanical, and biological measures with the minimal use of synthetic pesticides, under the name of integrated pest management, have been promoted. These combinations include high and low temperatures; the removal of dockages; and the application of pheromones, diatomaceous earth, and natural compounds from various plants, as well as inert gases, predators, and parasites. A ban of any synthetic pesticide usage is currently being considered, which emphasizes the fact that protection should only be performed by measures that do not leave harmful residues. However, the facts show that the application of physical, mechanical, and/or biological measures, besides the fact that they are not necessarily efficient, is very demanding because more knowledge and experience is required, as well as better equipment, greater financial investment, and awareness raising not only for agricultural producers and storage keepers, but also for consumers. In order to use these measures, which are less hazardous to humans and the environment, it is necessary to adapt regulations not only to speed up the registration protocols of low-risk pesticides, but also to prescribe criteria for placing agricultural products on the market, as well as quality standards, i.e., the permitted number of present insects, in addition to their parts in certain types of food. Additionally, we should be aware of control measures for protecting novel food and other non-traditional foods. It is important to continue to combine different protection measures, namely integrated pest management, until all of the other new procedures that must be carried out during the period of storing cereals and other products are clear, in order to ensure the best quality of final products for consumers.

## 1. Introduction

Storing grain in warehouses is important for preserving the quantities produced and providing a sufficient amount of food for the population. Well-preserved cereals are vital for producers for obtaining a quality product in accordance with the properties of their culture, and for consumers to receive food products of a good quality. For the cultivation of crops in the field, farmers need to make significant financial investments and put in many hours of work; however, after the goods enter a warehouse, concerns regarding the loss of quality gradually decrease, so losses can occur and the quality and quantity of stored goods can also decrease.

In this paper, we want to point out the problems that may arise during storage and the possibilities and impossibilities of solving them.

## 2. Trends in Agriculture

The European Green Deal was published in the European Union (EU) in December 2019, describing how Europe will become the first climate-neutral continent by 2050 and how to boost the economy, improve health and quality of life, protect nature, and not neglect anyone. The European Green Deal covers all economic sectors, including agricultural, chemical, and information and communication technologies. Significant investments will be needed to achieve the objectives of the European Green Deal [1].

At the end of May 2020, the European Commission presented the Biodiversity Strategy that will last until 2030, in order to achieve its ambitions in terms of climate and the environment. The goals, among others, are to protect areas with a very high biodiversity and climate value; restore degraded ecosystems across the EU; improve knowledge, financing, and investments; and better respect nature, demonstrating that the EU is ready to lead in this field [2]. At the same time, a “Farm to Fork” strategy for sustainable food production and proposals for pollution-free Europe have been published, especially for pollution caused by the use of pesticides in agriculture, which contributes to soil, water, and air pollution. The goal of the “Farm to Fork” strategy is to take measures to reduce the use and risk of chemical pesticides by 50% by 2030 and to reduce the use of more dangerous pesticides by 50% by 2030.

The use of chemical pesticides in agriculture contributes to soil, water, and air pollution and the loss of biodiversity and can harm non-target organisms and human health. The European Commission has already established a uniform risk indicator to quantify progress in reducing pesticide-related risks. Based on this indicator, it can be seen that in the last five years, the risk of pesticide use has decreased by 20%. There will also be a revision of the directive on the sustainable use of pesticides and improvement of the provisions on integrated pest management and the promotion of the greater use of safe alternative ways to protect crops from harmful organisms.

The European Commission will also facilitate the placing of pesticides containing biologically active substances on the market and will improve the environmental risk assessment of pesticides. It will take measures to shorten the duration of the pesticide approval process in the member states [3].

It is important to know that the adopted strategies are not legally binding, but that on the basis of the recorded situation in the EU member states, regulations must be adopted that will determine the implementation of the plans stated in the strategies.

The “Farm to Fork” strategy aims to reduce the use of pesticides and risks in outdoor agricultural production, as well as to improve the application of integrated protection measures, while the use of silos and agricultural warehouses for storing agricultural products is not even mentioned as these methods are considered irrelevant. However, integrated protection measures in warehouses are not a rarity but a rule and are important in the storage of agricultural products. The list of different goods stored in warehouses is as long as their uses as raw materials, directly for food, for processing, or for seed. There are not only differences in this, but also in the types of storage, the procedures occurring during storage, and the types of pesticides used to control pests of stored agricultural products. Our aim is to indicate how cereals stored in silos or warehouses are handled and what measures are taken to preserve them, to describe the situation regarding pesticides, and to point out the problems that occur during application and the possibility of applying substitutions.

## 3. Storing Cereals

During storage, the goal is to preserve the stored goods both quantitatively and qualitatively. If we exclude losses caused by natural changes such as the gradual drying of grain and respiration and other processes that contribute to a reduction of the quantity and quality, the most important aim is to protect goods from losses caused by microorganisms and storage fungi and pests.

Monitoring the change in heat of stored agricultural products and sampling is not always carried out systematically, which makes it difficult to know the true condition of stored cereals. In order to gain insight into the condition of stored cereals, it is necessary to take samples regularly, check the temperature of stored goods, and record data. Continuous temperature monitoring within stored grain is relatively easy using thermocouples. When an increase in temperature is observed, it can be assumed that the insect population is present or the existing one has started to grow and be more active. The present population of storage fungi can also cause an increase in the temperature of stored goods. However, by using available techniques, it is not easy to detect small insect or fungi populations [4].

Temperature is one of the key environmental factors affecting the physiological, life history, behavioral, and population processes of arthropods [5]. The largest increase in insect population occurs at optimal breeding temperatures ranging from 25 to 32 °C. Development is slowed down at temperatures ranging from 13 to 24 °C and 33 to 35 °C. Lethal temperatures are below 13 °C and above 35 °C, when insects stop feeding, development is slowed down, and insect death occurs. The more extreme the conditions, the faster the insects die [6]. However, the mentioned relationship between temperature and the developmental rate varies among different strains, species, and higher taxa [7]. In a recent review, Stejskal et al. [5] presented an extensive approach on stored pests and temperatures, with an emphasis on the lower development thresholds that define temperature zones for safe storage. Imura [8] estimated the insect lower temperature thresholds (LT) and degree-days (DD) required for egg to adult development for 51 stored products. The lowest LT = 6.9 and the highest DD = 1551.5 were obtained for *Hofmannophila pseudospretella* (Stainton), whereas the highest LT = 19.8 °C (lab) to 21.5 °C (Ghana) and the lowest DD = 319.4 to 286.6 were obtained for *Latheticus oryzae* (Waterhouse). Accordingly, for *Sitophilus granarius* (L.), LT = 10.5 °C and DD = 517.6, whereas for *Sitophilus oryzae* (L.), LT = 13.5 (13.6) °C and DD = 466.7 (422.1). The estimated values for *Tribolium confusum* (Jacq. du Val.) were LT = 17.8 (17.9) °C and DD = 334 (415.2), whereas those for *Tribolium castaneum* (Herbst) were LT = 10.5 °C and DD = 517.6.

Accordingly, it is best to store cereals at lower temperatures. However, due to environmental conditions, it is not always possible to achieve appropriate low temperatures.

In addition to the storage temperature, grain moisture is also important. It is certainly important to bring the driest grain into the warehouse, but if it is necessary to dry it due to a high level of grain moisture, this drying process should be done by gradually lowering the moisture; otherwise, sudden drying can cause the cracking of grain, reducing the quality and creating optimal conditions for storage pests and fungi. A grain moisture value from 13 to 14% is optimal for storing wheat and corn [9].

### 3.1. Storage

Cereals are stored in different types of storage. Large quantities of tens of thousands of tons are usually stored in concrete silos arranged in a row, and significant quantities are also stored in metal silos and warehouses. Smaller farms use smaller capacity silos, usually made of metal, which are adapted to the size of the farm and the quantities produced and required. On small farms, cereals are stored in various containers or handy warehouses according to production for their own needs.

It is important that grain is stored in a properly constructed warehouse. This means that, during storage, there is no wetting from the breathing of the grain, there are no openings in the concrete walls through which insects or rodents can enter, and that no wetting occurs during precipitation. Additionally, the grain temperature close to the wall changes with the season [10]. Apart from in exceptional cases, silo compartments need not be specially protected for impermeability, such as through treatment with carbon dioxide or phosphine.

### 3.2. Risks Associated with Storage Pests

Stored-product insects can be found in the bulk storage of raw commodities, in processing facilities such as flour and feed mills, in food-manufacturing facilities and bakeries, and in all other structures employed for food product storage or transport [11]. When it comes to food safety and quality, high standards are priorities in most countries. Although Europe has mandated a zero tolerance for insect fragments in food, in real conditions, this is hardly fulfilled. According to the EU database named the Rapid Alert System for Food and Feed (RASFF), recent analysis [12] revealed that the top three reported foreign materials found in agricultural food products were arthropod pests (54.6%), glass fragments (17.4%), and metals (11.5%). Estimated postharvest losses caused by stored-product insects range from up to 9% in developed countries to 20% or more in developing countries [13]. Risks associated with storage pests are diversely manifested as direct physiological effects and indirect effects [14]. The most important direct effect is associated with the contamination of food by arthropod fragments, which can be carcinogenic or allergenic. Stored-product pests, mites, and insects produce a variety of chemical compounds that cause allergic reactions in humans and domestic pets [15,16]. Indirectly, stored pests change the moisture content and temperature of stored food, developing optimal conditions for the growth of pathogenic microorganisms [17], or they host and transmit microorganisms, including antibiotic-resistant strains [18,19].

The fact that stored pests affect the food quality and adversely affect human health at a deeper level means that extensive management within the integrated pest management (IPM) programs is a key element required for their control.

## 4. Integrated Protection Measures

Integrated protection measures give preference to mechanical, physical, and biological measures, but if the required effectiveness is not achieved, some of the permitted insecticides must be used. During application, it is important to take care that the combination of pest control measures is both economically and environmentally justified, and that the risk to humans and the environment is the lowest possible.

Today, there is more and more talk about banning any use of synthetic pesticides and ensuring that protection is only produced by applying measures that do not leave harmful residues. However, the question of what to do if these non-pesticidal measures cannot ensure an adequate effectiveness against pests arises. Who will bear the responsibility and consequences for the quantities and quality of goods in trade and processing?

The facts show that the application of mechanical, physical, and/or biological measures is very demanding because it requires more knowledge, better equipment, greater financial investment, and raising awareness not only among storekeepers and grain processors, but also among consumers.

### 4.1. Mechanical Measures

These measures are performed by employees in the silo or warehouse, and include cleaning the empty storage space of dust, insects, and various residues from previous commodities. It is best to use vacuum cleaners for this, and where possible, wash the surfaces. Cereals in the warehouse are often moved from one place to another, i.e., from one silo compartment to another, and when unloading, the impurities are removed by aspiration. This measure can reduce the number of damaged grains, remove insects from the grains, and eradicate other impurities more easily from healthy grain. Unfortunately, this procedure is not able to remove insects that live in the grain, and this can cause a problem during transport, as it is possible to see live insects emerging from the grain that have not been removed by aspiration at the final destination. Of course, the question of whether the buyer will accept to take over such goods or will return them to the sender or will request the performance of a countermeasure at the expense of the supplier at the place of delivery arises. Good sanitation measures generally represent a starting point for the success of integrated pest management programs. Morrison et al. [20] found that decreased sanitation negatively affected the efficacy of most tactics examined, with a mean 1.3–17-fold decrease in efficacy under poorer sanitation compared to better sanitation. Therefore, poor sanitation can result not only in refugia of insects in grain bins providing re-infestation, but can indirectly decrease the efficacy of biological measures [21], chemical control tactics [22], and modified atmosphere regimes [23].

### 4.2. Physical Measures

Physical measures that can be performed to protect against pests on stored cereals take into account different ways of cooling.

If the heat of the goods exceeds 18 °C, it is necessary to perform cooling by inserting cold air from the environment or cooled air or to transfer cereals from one silo compartment to another. It is important to maintain a low temperature and relative humidity in the storage and to ventilate when the air is dry and cold [24]. The cold air blower installed in the silo must be switched on until the heat of the goods is reduced at the top, i.e., until cold air penetrates to the top. Maintaining low temperatures in a warehouse or silo is essential because it slows down the development and activity of pests of stored cereals or storage fungi that are present [25]. Chilled cereals remain cold, even at higher outdoor temperatures, due to the poor thermal conductivity of cereals. Switching, which is most often done in silos, does not contribute to significant cooling due to the poor thermal conductivity [6]. If it is possible to choose between switching from compartment to compartment and injecting cold air with cooling devices, it is better to introduce cold air into the grain and reduce the damage. Without considering the difference in energy consumption for each procedure, switching has the advantage that aspiration can be switched on and various impurities, damaged grains, and insects can be removed. The performance of the cooling method significantly depends on an insect’s cold tolerance. Even life stages of individual species vary in susceptibility to cold [26]. It has been proven that some stored product insect pests were mobile at low temperatures (2.5 °C), while other species could not move until temperatures reached 10 °C or higher [27]. Another relevant factor of cooling grain as a physical method is the temperature decrease rate. Jian et al. [27] concluded that when grain inside bins is cooled rapidly, the movement of insects can be stopped at a higher temperature than that when grain is cooled gradually. The temperature fluctuation allows an opportunity for insects to adapt to low temperatures [27,28].

Heat treatment is another possibility of physical measures for insect control during storage. It can be implemented through different treatments: As solar heating; water-based and atmospheric heating; steam treatment; flame treatment; forced hot air treatment; electric field treatment; and high-temperature-controlled atmosphere treatment [29]. The target air temperature for effective disinfestation should be at least 50 °C [30,31]. Factors that affect success in heat treatments are the insect species [32], treatment duration [33], and life stages [34]. Additionally, several other aspects should be taken into consideration. Insects inside kernels are more protected than individuals outside the kernel. Therefore, with the treatment of 105 °C hot air, *S. oryzae* adults outside wheat kernels showed about two times greater mortality when compared with young adults inside wheat kernels [35]. The grain quality, especially the seed germination rate, could be greatly affected by high temperature treatment. The treatment required to reach 100% mortality of insects inside kernels caused a 20% drop in germination in steam and 81% drop in hot air [35]. Another relevant issue is maintaining a constant rate of temperature during the whole treatment exposure period regarding the specifics of a commodity’s nature and industry or storage facilities. The authors Porto et al. [31] developed a method to improve the effectiveness of heat treatment for insect pest control in flour mills by thermal analyses and temperature trend models, with specific attention being paid to surface temperatures of thermal bridges as heat treatment weakness points. Furthermore, there have been some advances in heat treatment techniques. For instance, the radio frequency as a dielectric heating-based technique is considered to have an improved efficacy compared with traditional heating techniques [36] due to its fast, volumetric, and deep penetration heating characteristics [37].

### 4.3. Application of Diatomaceous Earth

Diatomaceous earth (DE), belonging to a group of inert dusts, has recently been considered as one of the major alternative protectants for the grain industry.

DE is a rock mineral made of fossilized single-celled algae called diatoms. The main component and active ingredient of DE is amorphous silicon dioxide (70–90%) [38,39]. Due to the particles having very small inner pores, DE has a physical mode of action against insects. It adheres to the insect body and absorbs the protective waxy layer of the insect cuticle, damaging it by abrasion. Consequently, insects lose body water, leading to death [40].

A low toxicity to mammals, efficient insecticidal activity, and long-term activity are considered to be the main advantages of using DE in stored product protection [41,42,43]. It is suitable for an integrated pest management strategy in the grain and food industry as a grain protectant against stored product insects. Losic and Korunic [38] gave a comprehensive overview of DE application modes. Wet spraying (as DE slurry) and the blowing or dusting of powder to grain (as dry DE) are common methods of application. Spraying is easier to apply and creates a less dusty atmosphere, which ensures safer handling for workers, but the efficacy can be reduced [44]. Dry treatment of DE is suitable for the protection of stored facilities and grain handling equipment, and with the prior physical cleaning of grain residues, preserves safe and clean storage for new commodities after harvesting.

However, there are some limitations of using DE that hinder its wider usage in storage of a larger capacity or in the milling industry. These are related to the adverse effects on the physical and mechanical properties of grain (impact on the bulk density and grain flow) [45] and abrasion of handling machinery [38]. Due to these issues, DE can hardly be accepted for direct mixing with grain by the grain industry. Some prospective usage is possible for structural treatments and direct mixing with grain on small farms [46] or for direct mixing with feed in feed production [47].

The development of enhanced DE formulations is of great importance, in order to achieve the same or higher level of efficacy, but with a lower amount of DE, which would have a minimal effect on the bulk density and grain flow ability. Some of the promising solutions are mixing DE with low toxicity synthetic insecticides [48,49,50] and other reduced-risk methods with insecticidal activity, such as grain cooling [51], heat treatment [52], combination with entomopathogenic fungi [53,54], and a mixture with plant extracts [43,55,56].

### 4.4. Biological Measures

Much research has been performed on the possible use of biological measures over the past years. Most of applications have been used against stored product moths in bakeries, food processing industries, retail trade, and households and against weevils on farms, but 50% of this type of application has involved the control of Pyralidae moths [57].

Biological measures include the use of predators and parasites. Among the best known are the predatory species *Xylocoris flavipes* (Reuter) and the parasitoids *Anisopteromalus calandrae* (Howard), *Habrobracon hebetor* (Say), and *Holepyris sylvanidis* (Brèthes), which successfully reduce the population of various storage pests, e.g., *Tribolium* spp. *Callosobruchus maculatus* (F.) and others in small quantities of goods. The successfulness of biological measures significantly depends on the olfactory host location and host preference. Therefore, research is evolving towards the discovery of stimuli responsible for attracting predators and parasites in the host location [58,59]. In contrast to predators and parasitoids, which are mostly host specific, insect pathogens have a broad spectrum of hosts [60]. Therefore, a promising microbial control strategy includes entomopathogenic fungi (EF), which induce disease symptoms and cause lethal infections in insects [61]. In addition, fungus develops in the insect cadaver and sporulation occurs on the external part of the body of the dead host, reinterring more inoculum for the next infections [62]. The most studied fungal species for the control of stored product insects are *Beauveria bassiana* (Balsamo) Vuillemin (Ascomycota: Hypocreales) and *Metarhizium anisopliae* (Metschnikoff) Sorokin (Ascomycota: Hypocreales) [63]. In addition to the successfulness of their application, it is evident that their pathogenicity and virulence vary remarkably among the host species and the life stage of the target pest [64,65]. Furthermore, abiotic factors are crucial for the success of EF in controlling post-harvest insects, with temperature and humidity as the key elements for ensuring their efficacy [66]. However, both the grain type and the isolates are equally important factors for ensuring the efficacy of EF [67]. Batta and Kavallieratos [68] presented a comprehensive review of possible improvements of EF application in storage systems. They highlighted the importance of the isolation of new and effective strains of EF, discussed the types of EF formulations required to achieve a constant and higher control efficacy, and proposed the possibility of combinations of EF with other products i.e., DEs, chemical insecticides, natural products, and natural enemies.

One of the least investigated biological control agents for the control of stored product insect pests are entomopathogenic nematodes. There are several advantages of their application, i.e., safety for humans, mammals, and other non-target organisms [69]; the potential of application with conventional spraying technology [70]; and compatibility with many types of pesticides [71]. The nematode species tested against stored product insects exclusively belong to the genera *Steinernema* and *Heterhorhabditis* [72]. On the other hand, dry conditions that prevail in most storage facilities negatively impact the efficacy of nematodes, which affects their short-term post-application survival and persistence [72]. In order to protect nematodes from biotic and abiotic stress, significant improvements have been achieved. For that purpose, different encapsulation methods, i.e., alginate-based entomopathogenic nematode capsules, provide an improvement of their efficacy [73,74].

However, there are still unknowns, such as the potential of beneficial organisms, biology, and behavior, as well as the reasons why certain species are attractive to them [75]. Additionally, an important problem is the finding of predators or parasites, as many who will buy a product that contains these beneficial organisms will not want to take/buy them because they contain living organisms, and they do not care what they are, but simply do not want them on the goods. Therefore, educating customers so that they can distinguish between harmful and beneficial organisms should be conducted, as well as raising awareness that such goods are not treated with pesticides and do not contain their harmful residues.

In accordance with the above, it is necessary to improve the equipment of storage facilities by installing appropriate cooling devices and educating employees on the continuous monitoring of stored goods by taking samples and the timely cleaning of stored goods and cooling. The application of DE is more convenient for small storage facilities than on a bigger scale, but one must be aware that there could be a problem with the bulk density and this is not accepted everywhere equally. If it is decided that biological control measures will be implemented, it is important to know for which goods, quantities, and types of storage these are applicable, and it is also necessary to educate employees and know the pests occurring on the goods.

### 4.5. Pesticide Treatment in Storage

For decades, the use of various synthetic pesticides has been the basis for the proper and long-term storage of insect- and mite-free cereals. However, as the negative aspects of synthetic pesticides have been discovered, efforts are being made to apply them less frequently, and integrated protection measures are increasingly being taken. However, for now, the use of pesticides is still an indispensable method of the pest control of stored agricultural products in many cases.

Empty space and stored goods are treated in the warehouse.

#### Treatment of Empty Storage Space

Before treatment with pesticides, the storage area must be cleaned of dust, the remains of goods, and even insects as best as possible, in order to achieve the required efficiency. This is especially important when spraying surfaces with insecticides because the dust absorbs the applied insecticide and the required effectiveness can then not be achieved for the present insect population or that which comes with the cereals to the warehouse.

The choice of products that can be used to control storage pests is small. After many years of usage, the use of malathion was stopped, primarily due to the appearance of resistance, and the use of dichlorvos was then banned. Today, in practice, only pirimiphos-methyl is actually used to treat empty space. The biggest problem for experts was the ban on the use of the fumigant methyl bromide, which was applied to various goods with a relatively short exposure. Phosphine and, more recently, sulfuryl fluoride, have been proven to be the best substitutes, and carbon dioxide is one of the most environmentally friendly. Table 1 lists the names of the active substances and the expiry date of the authorization, as well as the types of application.

According to the data in the table, it can be seen that the permits expire after a short period of time, and if the permits are not extended, many damages and losses in quality and quantity can be expected during the storage of cereals.

If the reasons for non-renewal of the authorization are not related to health or environmental protection, a delay period of up to 6 months is provided for sales and distribution, and an additional maximum of 12 months for the disposal, storage, and use of existing pesticide stocks.

If the authorization is not renewed due to concerns for human or animal health or the environment, the pesticide is withdrawn from the market immediately [77].

Protectants are insecticides that are applied directly to the grain of cereals, and are important because the treatment is most often conducted at the beginning of storage at the entrance of the grain after cleaning and when there is the least dust, which reduces the efficiency. The purpose is to provide preventive protection which ensures effectiveness against pests for several months. The insecticidal efficacy only applies to insects found outside the grain. In situations where a grain insect population is present, fumigation is more important and effective.

Carbon dioxide of ≥99.9% purity is registered for application as a fumigant in a gas-tight silo unit without circulatory fumigation, bulk storage, a granary, a Pex-pressure chamber, and a Carvex-pressure chamber. For application outside pressure chambers, in addition to the applied amount/concentration, the temperature is important for obtaining a fast and proper efficacy, whereas in pressure chambers, the pressure is important [5]. The application itself is not easy because it can be applied from a tank and is introduced into the mass of dormant grain from the top, where it is lowered due to its weight, or it is applied as dry ice at the top of the silo. The application ensures efficiency on insects. Fumigation in the chambers under high pressure is inherent in the treatment of high-value goods such as herbs and spices. The wider use of carbon dioxide on cereals in the silo has not survived due to the large amounts required for application and good sealing, i.e., silo impermeability and long exposure. At the same time, application in chambers under high pressure is relatively well-accepted; although the initial investment for procurement is considerable, the relatively rapid completion of fumigation amortizes the initial investment. The required efficiency can be achieved for both insects and mites.

Sulfuryl fluoride may only be used in empty silos or storage facilities to control storage pests. If there were cereals or other goods in the warehouse during fumigation, they must not be given to humans or animals for food. Due to the impossibility of treating grains in the warehouse, the wider use of sulfuryl fluoride has not occurred.

Phosphine is a gas that is released from aluminium (formulation-tablets, pellets, and bags) or magnesium phosphide (formulation-tablets and pellets). Although, in reality, phosphine is effective against different pests from a legislative point of view, aluminium phosphide and magnesium phosphide are understood as two different active ingredients and were included separately in Annex I of the Directive 91/414 EC. After inclusion in Annex I, there is a procedure for registration of the product/formulation [76]. Phosphine is also commercially available from generators in pressurized gas cylinders as a non-flammable 2% mixture in liquid carbon dioxide and as a 1.7% mixture in compressed nitrogen [78]. Such a product/formulation has to undergo the same procedure as others. Phosphine is widely used on various products, such as cereals and oilseeds in silos, but also various goods, such as almonds, cocoa in bags, or tobacco in boxes. Phosphine is effective against all developmental stages of storage pests. It is important that, due to its good penetration, it also suppresses insect infestation hidden in the grain. There are some registrations of products releasing phosphine in which it is explicitly mentioned: “Pellets/tablets or their spent residue must not be allowed to come into contact with food or feed” [79]. In accordance with the food law [80] “food means any substance or product, whether processed, partially processed or unprocessed, intended to be, or reasonably expected to be ingested by humans”, it can be understood that grain and similar products are not allowed to be treated with currently available products releasing phosphine. The reason for this are the rests/remains of the product that remain in stored cereals and oilseeds after phosphine fumigation. If suitable solutions are not found in the production of pellets and tablets, as well as the suitable cleaning of grains, which remove the remains of formulation after phosphine release, the danger is that it will not be possible to treat cereals and similar products and the final result will be their poor quality. However, in the same registration, it says, “Other specific restriction does not apply in the following circumstances: (a) Treated unprocessed food or feedstuffs are destined for export outside of the EU and (b) the commodity is treated under contract from the destination country and the sanitary/phytosanitary requirements of the importing country specifies that direct insertion of pellets/tablets is required”. However, it is difficult to understand why products for export from the EU might be treated. The other relevant point of phosphine usage is that due to the development of resistant populations among stored insects, the use of phosphine becomes questionable in some cases. From the global perspective of resistance to phosphine in insect pests of stored products, there have been reports of control failures in several countries, indicating evidence of higher or stronger levels of resistance in some species [81]. The most extensive resistance data are available for Australia, referring to strong resistance for *Rhyzopertha dominica* (Fabricius), *S. oryzae*, *T. castaneum* [82], and *Cryptolestes ferrugineus* (Stephens) [10]. In Asia, strong resistance has been reported in populations of *R. dominica* [83], *S. oryzae* [83,84], *C. ferrugineus* [85], and *Trogoderma granarium* (Everts) [86]. In North America, there is evidence of an increase in the frequency of phosphine resistance for *R. dominica*, *C. ferrugineus*, *Plodia interpunctella* (Hübn), and *Lasioderma serricorne* (F.), ranging from 80 to 100% for some species [87,88,89,90]. Until recently, there has been little or zero information on phosphine resistance in European populations of storage pests. However, a recent increase in the number of published papers indicates that phosphine resistance may have a strong impact on chemical control in European grain stores and warehouses. Greek researchers conducted extensive research [91] in grain storage facilities across Greece and detected resistance in populations of several species, suggesting that the field test kit named the Detia Degesch Tolerance Test Kit (DDTTPK) can be used to determine resistance to phosphine in the field, before the initiation of fumigations to disinfest stored commodities. Recently, the first evidence of resistant strains of *S. granarius* and *T. castaneum* in the Czech Republic was reported [92]. In order to avoid or prolong the development of resistant populations of stored pests, researchers have been focusing on addressing gaps in and control failures during the protocols of phosphine fumigation. Aulicky and Stejskal [93] conducted field validation of spot-fumigation with phosphine and concluded that, despite positive results, undetected pests were treated inefficiently, and such exposure to low or sublethal gas doses poses a risk for an increase in phosphine resistance. Practical solutions are being developed for phosphine treatments in mills, in order to maintain complete control of all life development stages of *T. confusum* and *C. ferrugineus*, with a special focus on eggs [94]. More field studies like these are needed to provide data to establish recommendations about optimizing phosphine fumigation and implementation of the integrated approach of control measures.

Table 2 provides an overview of the measures implemented as part of integrated measures for the protection of stored agricultural products, when certain measures are taken, how they are implemented, and the purpose of implementation. It is stated who can implement certain measures, since there are significant differences from employees to authorized professionals for certain groups of pesticides. Some disadvantages and advantages are listed as they are present in each of these measures.

In order to be able to use measures that are less dangerous for people and the environment, it is necessary to adjust regulations not only to speed up the registration of low-risk pesticides, but also to prescribe criteria for placing products on the market and quality standards, i.e., how many insects and their parts present in certain types of food might be allowed or not allowed.

It is certainly important to continue to implement a combination of different protection measures, i.e., integrated protection measures, until all the procedures that must and can be performed in warehouses during the storage of cereals are properly known.

### 4.6. Botanical-Based Pesticides

Botanical pesticides are currently recognized as biodegradable and ecologically-friendly and are widely supported in organic agriculture systems. Because the active ingredients degrade quickly in soils and under UV light, their impacts on non-target animals, such as pollinators and natural enemies, as well as consumers, are often trivial [29]. Many botanicals are based on extracts from herbs and spices, and are thus categorized as “generally regarded as safe”. However, their modes of action are diverse and their bioactivity is often expressed through mixtures of compounds, which can be seen from different perspectives. One is the opportunity to avoid the development of resistant pest populations when applying pesticides of different modes of action. The other perspective is that pesticides mixtures of different compounds are challenges to regulatory standards, where pesticide regulations are generally designed for synthetic pesticides that contain a single, highly concentrated, and persistent compound. There are numerous published papers which have recorded the activity of different plants on stored product insects. When mixed with stored grains, leaf, bark, seed powder, or oil extracts of plants reduce the oviposition rate and suppress the adult emergence of stored product insects, and concurrently reduce seed damage rates [95,96,97,98]. Botanical insecticides mostly include ryania, rotenone, pyrethrin, nicotine, azadirachtin, and sabadilla [99], though there are limited commercially available products based on plant essential oils (EOs) or their isolated compounds (Table 3).

Despite their main advantages of being eco-friendly, easily biodegradable, nontoxic to non-target organisms, and locally available, botanicals are not used as pesticides on a large scale. One of the main reasons for this is their physical properties, such as their high boiling point, high molecular weight, and very low vapor pressure [101], which is the cause of their short persistence and lower toxicity in comparison to synthetics. Additionally, the aromatic nature of EOs and odor retention in treated stored goods further restrict application to only empty storage or goods in which the presence of odors represents no limit for usage or processing [102]. Despite that, regulatory approval remains the greatest barrier to the implementation of botanical insecticides, especially in the European Union, where it now takes approximately four years from data submission to approval [103]. However, due to insect resistance to conventional insecticides and regulatory limitations and bans of major active ingredients, there is an increasing demand for alternative solutions such as botanical pesticides. Furthermore, the awareness of and need for safer food without pesticide residues could represent new opportunities for botanical pesticides to become a more important solution for crop protection. However, to overcome those obstacles, there is a great challenge in searching for new technologies that would allow wider application and increase the efficacy of botanical-based pesticides. For example, plant biotechnology presents potential with the genetic manipulation of some field crop species, in order to produce large quantities of natural compounds originally isolated from other plant species [104]. Other alternative prospects are possible through the implementation of nanotechnology.

### 4.7. Nanopesticides

Nanotechnology is recognized by the European Union as one of the six technologies vital for global sustainable development [100]. Nanoparticles can be classified as aggregated ultra-fine particles measuring 1 to 100 nm or less, having specific properties by which they differ from particles of the same chemical composition, but outside the nano size scale [105]. Nanoparticles can be formulated as emulsions, suspensions, polymeric plates, and gels [106] or act in the form of capsules based on silica gels, chitosan, sodium alginate or polyethylene glycol [107], silver, aluminium, zinc, or copper oxide. Nanopesticides represent an emerging tool for the integrated pest management approach that could provide an increased efficacy, durability, and reduction in the current amount of active ingredients used [108].

According to Losic and Korunic [38], there have been several studies exploring the use of synthetic silica and fragmented DE particles in the nanometer range, showing that they have certain advantages for use in insect control. Significant improvements in performance were achieved with formulations based on DE and aerogels with nano-size dimensions, applied as a dust or spray of suspended DE in water. Efficient activity against tested insect species was achieved with low dosages (100 ppm against *C. ferrugineus* and 300 ppm against *T. castaneum*) and concurrently had the lowest possible effect on the physical properties of grains. A study [109] conducted with silica in the nano range indicated that the looseness and bulk density of the grain were not affected, even with the highest dose of 2000 ppm.

In order to resolve the problems related to EO application, nanotechnology found a significant role in nanoemulsion formulations. Nanoemulsions have advantages over conventional emulsions due to their small droplet size, which ensures their stability [110]. The range of droplet sizes is from 20 to 200 nm. A multitude of those droplets are highly dispersed in a nanoemulsion, which increases its surface area. Powerful characteristics of nanoemulsions are expressed through a higher activity [110], reduced hydrolysis, and volatilization of the active substances [111], leading to an increase of residual activity. The low interfacial tension of the droplets provides an increase of surface coverage when spraying and dispersing, as well as better penetration through a commodity [112]. A study on a series of nanoemulsions of neem oil proved that the LC_50_ decreased with the droplet size, which was explained as the increased uptake of smaller droplets [113]. A nanoemulsion of lavender EO showed higher and faster activity against *T. castaneum* and *S. oryzae* in treated wheat in regard to activity of the reference emulsion [114]. A similar enhanced activity of a nanoemulsion based on EO from two plant species (Fam. Labiate) with pulegone as their main constituent over a coarse emulsion was reported [115].

Since fogging and aerosol systems are increasing focus among the scientific community as alternative methods of fumigation in commercial food storage facilities [116], the authors Giunti et al. [117] suggested sweet orange EO nano formulation for application as cold aerosol as a promising method for the sanitation of production areas, warehouses, handling equipment, and production machinery against stored product pests. Depending on the formulation, nanopesticides can be applied directly as sprays and fumigants or as granular formulations [118]. Nonetheless, most of the results of nanopesticide application in stored product protection refer to laboratory tests. A major issue that remains to be addressed is the need for more detailed assessments in field conditions. Furthermore, due to potential toxicity concerns of nanomaterials, which are not standardized, not well-understood, or and not well-explored [119], risk assessment studies are essential before their usage.

### 4.8. Pheromones and Traps

Pheromones are registered in accordance with the regulation concerning the placing of plant protection products on the market [77] or regulation of biocidal products [120]. The use of pheromone traps is not as common in warehouses as it is outdoors or in green houses. Pheromone traps used in warehouses can be in the form of a “delta trap”, with a sticky insert or a sticky trap (plate). The pheromone trap contains a capsule of the synthetic pheromone that emits an odor that attracts the corresponding type of insect. This method determines the presence of insects in the warehouse. It can influence the decrease of the present population or be used as a control method. Although pheromones attract many individuals, it is difficult to expect that every individual in one storage space will be attracted and that this will achieve a complete reduction of the population, i.e., complete control of the species present. Their application is suitable in a monitoring program that shows the presence and possible need for the implementation of a control measure or as a method of determining the effectiveness of conducted control measures. Pheromones containing the scent of the female moth, such as *P. interpunctella*, *Ephestia* spp., etc., are most often used in warehouses and attract males, leading to mating disruption. The smell from the capsule interferes with the male’s ability to find females. The result is reduced mating and egg-laying by females. Trematerra at al. [121] found that in the second year of exposing pheromone traps, the population density of *P. interpunctella* and *Ephestia cautella* (Walker) decreased. When applying pheromones, it is important to know how many traps must be placed according to the space size and how long the smell is released. They can also be aggregation pheromones secreted by male beetles, i.e., Tenebrionidae, Cucujidae, etc. These pheromones attract both females and males and gather around the odor on the most commonly used sticky plate.

The use of pheromones is important in the program of integrated pest management measures and it is certainly important to work on research to find new attractants or repellents that could be used in warehouses of agricultural products.

## 5. Conclusions

At a time when there is much talk of reducing pesticide use and the implementation of integrated pest management, it is appropriate to consider how cereals in storage are handled and what measures have been taken to protect them, mainly against insects, but also other pests.

The storing of cereals is important to preserving the quantities produced and providing sufficient food for the human population.

Integrated pest management has been promoted combining physical, mechanical, and biological measures, as well as use of nanotechnology or pheromones. It has to be taken into account that many of these measures cannot completely control the insect or mite populations and are very demanding because they require more knowledge and experience, better equipment, greater financial investment, and awareness raising, not only for agricultural producers and storage keepers, but also for consumers.

It is important to continue to combine different protection measures during the period of storing cereals.

## Figures and Tables

**Table 1:** Active substances and dates of expiration of approval and type of treatment [76].

Active Substance	Expiration of Approval	Type of Treatment
Dichlorvos	1998	Protectant
Pirimiphos-methyl	31 July 2021	Empty storage
Methyl-bromide	2011	Fumigant
Carbon dioxide	31 August 2020	Fumigant for grains
Aluminium phosphide	31 August 2022	Fumigant for grains and empty storage
Magnesium phosphide	31 August 2022	Fumigant for grains and empty storage
Sulphuryl fluoride	31 October 2023	Fumigant for empty storage

**Table 2:** Measures applied in storehouses containing grains.

Measure	When It Is Undertaken	How It Is Implemented	Purpose of Application	Who Applies	Disadvantages	Advantages
Mechanical	During entry/exit from the warehouse and during storage	Cleaning	Removal of impurities, including damaged grains, insects, mites, chaff	Warehouse workers	It does not control insects or mites present	Cleans grain of impurities
Physical	During storage at elevated temperatures	Cooling	Cooling the heated grain mass	Warehouse workers	It does not control insects or mites present	Grain temperature reduction; slowing the development of insects or mites
	At the beginning of storage	Spraying/dusting of DE	Control of insect population	Professionals—contracted company	Dust and decrease of bulk density	Ecologically acceptable
Chemical	Preventive when entering the storage	Insecticide spraying	Protection against possible insect attack	Professionals—contracted company	Unnecessary application of insecticides and remaining insecticide residues	Ensuring long-term protection for several months
	Curative upon entering the storage or during storage period	Insecticide spraying	If a population of insects and mites is present outside the grain	Professionals—contracted company	Damage has already occurred and insecticide residues remain	Population growth and damage are stopped
	Curative when all developmental stages of insects or mites inside and outside of the grain are present	Gas application	Suppression of the present population	Authorized professionals for the application of gases—contracted company	Upon end of fumigation (gas action), i.e., after aeration, there is no insecticidal effect	It acts on all developmental stages inside and outside the grain
Biological	Curative, depending on the type of insects or mites present	Application of parasites or predators, depending on the type of insects or mites present	Control of insects or mites present	Trained warehouse workers or contracted company	On the grain remains the action of harmful or beneficial insects and mites. Possible presence of beneficial organisms	Control of insect and mite populations without residues
Application of pheromones and traps	At any time	Pheromone type depends on the insect species present or supposed to be present	Mating disruption, discovery of population	Trained warehouse workers or contracted company	It is not a control method	Help with confirmation of performed control measures. Shows presence of pests.

**Table 3:** Commercially available products based on botanical pesticides [100].

Active Ingredient	Application	Commercial Product	Company
Oil of neem (Azadirachtin)	Insecticide	Margosom^®^	Agri Life (Telangana, India)
		AZA-Direct R^®^	Gowan Company (Arizona, USA)
		Azera TM^®^	MGK (Minnesota, USA)
		Azamax^®^	UPL Ltd.a. (Campinas, Brazil)
		Molt-X^®^	BioWorks Inc. (NY, USA)
		Neemix 4.5^®^	Certis (Columbia, USA)
		Azatin XL^®^	OHP Inc. (SC, USA)
		NeemAzal T/S^®^	Trifolio-M (Lahnau, Germany)
		Fortune Aza 3%EC^®^	Fortune Biotech (AP, USA)
		Shubhdeep NeemOil^®^	King AgroFood (Haryana, India)
Essential oil of garlic (*Allium sativum* L.)	Insecticide	AjoNey^®^	I.H.N. (Mexico)
		EcoA-Z^®^	EcofloraAgro (Antioquia, Colombia)
		L’EcoMix^®^	
		CapsiAlil^®^	
*Citrus cinensis* L. oil (limonene and linalool)	Insecticide/repellent	Demize EC^®^	Paragon Professional Pes Control Products (Tennessee, USA)
		Prev-Am^®^	Oro Agri SA Ltd. (South Africa)
EO of thyme (*Thymus vulgaris*)	Insecticide/repellent	EcoVia WD^®^	Rockwell Labs Ltd. (North Kansas City, USA)
Rotenone	Insecticide	5% Rotenone ME^®^	Bejing Kingbo Biotech Ltd. (Beijingm, China)
		Rotenone Dust^®^	Bionide Products Inc. (NY, USA)
Nicotine (*Nicotiana tabacum* L.)	Insecticide	Nico Dust^®^	Nico Orgo Manures (Gujarat, India)
		Nico Neem^®^	
		10% Nicotine AS^®^	Bejing Kingbo Biotech Ltd. (Beijing, China)
Carvacrol/EO of oregano (*Oreganum vulgare*)	Insecticide/animal feed supplement	By-O-reg+^®^	By-O-reg+ (SD, USA)
